# Detecting Tomato Leaf Curl New Delhi Virus Causing Ridge Gourd Yellow Mosaic Disease, and Other Begomoviruses by Antibody-Based Methods

**DOI:** 10.3390/plants12030490

**Published:** 2023-01-20

**Authors:** Priya Naganur, Kodegandlu Subbanna Shankarappa, Raghavendra K. Mesta, Chilakalapudi Durga Rao, Venkataravanappa Venkataravanappa, Midatharahally Narasegowda Maruthi, Lakshminarayana Reddy C. Narasimha Reddy

**Affiliations:** 1 Department of Plant Pathology, College of Horticulture, University of Horticultural Sciences, Bengaluru 560065, Karnataka, India; 2 Department of Plant Pathology, College of Horticulture, University of Horticultural Sciences, Bagalkot 587101, Karnataka, India; 3 Department of Biology, SRM University, Mangalagiri Mandal, Neerukonda, Amaravati 522502, Andhra Pradesh, India; 4 Division of Crop Protection, ICAR-Indian Institute of Horticultural Research, Hessaraghatta Lake PO, Bengaluru 560089, Karnataka, India; 5 Agriculture, Health and Environment Department, Natural Resources Institute, University of Greenwich, Medway Campus, Chatham, Kent ME4 4TB, UK; 6 Department of Plant Pathology, College of Agriculture, University of Agricultural Sciences, GKVK, Bengaluru 560065, Karnataka, India

**Keywords:** begomovirus, coat protein, ELISA, Geminiviridae, immunocapture assays, ridge gourd, tomato leaf curl New Delhi virus (ToLCNDV)

## Abstract

The incidence and severity of begomovirus diseases have been increasing around the world recently, and the ridge gourd [*Luffa acutangula* (Roxb.) L.] is the latest example of a crop that has become highly susceptible to the outbreak of the tomato leaf curl New Delhi virus (ToLCNDV, genus *Begomovirus*) in India. Accurate diagnosis of causal agents is important in designing disease management strategies. In this study the coat protein (CP) gene from a ToLCNDV-Rg ridge gourd isolate was used to produce polyclonal antibodies (ToLCNDV-Rg-CP-PAb) in a rabbit. The antibodies successfully detected a 30.5 kDa ToLCNDV-Rg-CP in extracts of symptomatic ridge gourd leaf samples by several assays, such as Western Blotting (WB), Dot Immuno Binding Assay (DIBA), Direct Antigen Coating Enzyme Linked Immuno Sorbent Assay (DAC-ELISA), Immuno Capture Polymerase Chain Reaction (IC-PCR), and Immuno Capture Loop-Mediated Isothermal Amplification (IC-LAMP) assays. However, none of the negative samples tested positive in either of the detection methods. Among all the methods tested, the immunocapture assay, IC-LAMP, was the most sensitive in detecting ToLCNDV-Rg. Furthermore, antibodies generated in this study also detected other commonly occurring begomoviruses in South India, such as tomato leaf curl Palampur virus and squash leaf curl China virus in cucurbits. Together, ToLCNDV-Rg-CP-PAb can be used for detecting at least three species of begomoviruses infecting cucurbits. The obtained antibodies will contribute to monitoring disease outbreaks in multiple crops.

## 1. Introduction

The ridge gourd [*Luffa acutangula* (Roxb.) L., *Cucurbitaceae*] is an important vegetable grown in India [1] and is used to prepare many culinary items including chutney and curries. It has both nutritive (carbohydrates, carotene, fat, protein, phytin, flavonoids, saponin, amino acids, vitamins, and minerals) and medicinal values (antidiabetic, antioxidant, abortifacient, and antifungal activity). Its hard fiber from mature fruits is used in industries for cleaning and scrubbing machines. Among the many biotic and abiotic factors affecting the ridge gourd crop [2], the yellow mosaic disease (YMD) of the ridge gourd caused by the tomato leaf curl New Delhi virus (ToLCNDV) (genus: *Begomovirus*) is emerging as a major problem to ridge gourd cultivation in India [3]. Begomoviruses are an important group of plant viruses belonging to the family *Geminiviridae*, which have circular single-stranded DNA (ssDNA) genomes encapsidated in characteristic twinned icosahedral particles [4]. The family *Geminiviridae* consists of 14 genera categorized based on their genome structure, mode of transmission by the insect vector, and host range [5], www.ictvonline.org, accessed on 1 October 2022. Begomoviruses are transmitted naturally by the insect vector whitefly, *Bemisia tabaci* cryptic species complex (Genn.). Due to its wide host range, the ToLCNDV has emerged as a major threat to the production of many crops belonging to diverse plant families *Acanthaceae*, *Apocyanaceae*, *Asteraceae*, *Caricaceae*, *Cucurbitaceae, Euphorbiaceae, Fabaceae*, *Malvaceae, Phyllanthaceae*, *Papaveraceae,* and *Solanaceae* worldwide [6,7]. Owing to its ability to adapt to new hosts, the host range of ToLCNDV is rapidly expanding [8]. Recently, a new strain of ToLCNDV has been reported to infect cucurbits, including the ridge gourd, causing RgYMD with up to 100% disease incidences in India [3].

Diagnosis of ToLCNDV is important to understand its epidemiology and disease management. Highly sensitive and efficient detection tools are required to identify resistant varieties and evaluate new germplasm in breeding programmes. Several tools (ELISA, PCR, NASH, IC-PCR, Real-time PCR, and LAMP) have been optimized for the detection and identification of ToLCNDV [9,10,11,12]. Commonly, ELISA has been used for routine virus detection owing to its simplicity, sensitivity, accuracy, and affordability. Monoclonal antibodies (MAbs) and polyclonal antibodies (PAbs) against different species of begomoviruses (Bean golden mosaic virus (BGMV), cabbage leaf curl virus (CabLCV), and tomato yellow leaf curl virus (TYLCV)) have been developed by using purified virus particles or recombinant virus proteins expressed in *Escherichia coli* system [13,14].

The main advantage of ELISA is that the virus titre in the test sample can be quantified; however, quantification is not necessary for most diagnostic work, where the goal is to determine the presence or absence of the virus in the test sample [4,10]. The DIBA is a simple, efficient, low-cost method, and the membranes can be stored for a longer period of time without losing sensitivity, and it has field-level applicability in virus detection surveys and is useful in epidemiological studies [10]. Although NASH is a valuable tool for DNA and RNA recognition, hybridization probes have several common drawbacks, including low selectivity under physiological conditions, low affinity for folded single-stranded RNA and double-stranded DNA, and the high cost of dye-labelled and chemically modified probes [9,10]. LAMP is more sensitive than all these methods, and the reaction would be completed in less than one hour. Further, it does not require thermal cyclers, and the amplification can be carried out with a water bath or heating block [11,12]. Immunocapture, followed by the detection of viruses using nucleic acid-based tools (PCR, LAMP), is a versatile, sensitive, and robust diagnostic technique. Application of these hybrid methods of virus detection in plants is particularly useful in species or tissues containing inhibitory substances [10]. Real-time PCR is used to quantify the concentration of the virus and offers greater sensitivity than conventional PCR, molecular hybridization, or serological methods [10].

The traditional method of begomovirus antigen preparation from infected plant tissue generally encounters many problems, including contamination with host proteins, low virus titre, presence of inhibitors, and instability of the particles, limiting their usage in sero-diagnostics [15,16,17,18]. To overcome these limitations various methods have been applied, including the use of recombinant DNA technology to express viral genes of interest in prokaryotic or eukaryotic cell systems for obtaining purified proteins, which can be used successfully in antibody production [19]. Protein folding, stability, and solubility are important for the biological activity of the recombinant protein for antibody production [20]. Most serological methods are based on CP because viruses are encapsulated by CP, and their detection reflects the number of virus particles in the test samples [21,22]. The sensitivity of the detection has been improved by combining serological methods with molecular methods, such as Immuno Capture Polymerase Chain Reaction (IC-PCR) and Immuno Capture Loop-Mediated Isothermal Amplification (IC-LAMP) [23]. LAMP will be carried with DNA extracted from normal DNA extraction procedures, whereas in IC-LAMP, the virus is captured on the PCR tube pre-coated with a specific antibody and ensures only viral nucleic acid is in the template. IC-PCR or IC-LAMP can be useful in detecting pathogens without nucleic acid extraction procedure or from plant species or tissues containing inhibitory substances hindering the quality of nucleic acid extraction [23].

In this study, novel PAbs were generated against the recombinant CP from ToLCNDV-Rg ridge gourd isolate and were used to optimize different diagnostic methods such as Western Blot (WB), Dot Immuno Binding Assay (DIBA), Direct Antigen Coating Enzyme Linked Immuno Sorbent Assay (DAC-ELISA), IC-PCR, and IC-LAMP for detecting begomoviruses in different crops. Our studies further revealed that the PAbs raised against ToLCNDV-CP can be used to detect other begomoviruses occurring in Southern India.

## 2. Results

### 2.1. Expression ToLCNDV- Rg-CP Gene and Purification of Recombinant Protein

**Figure 1:**
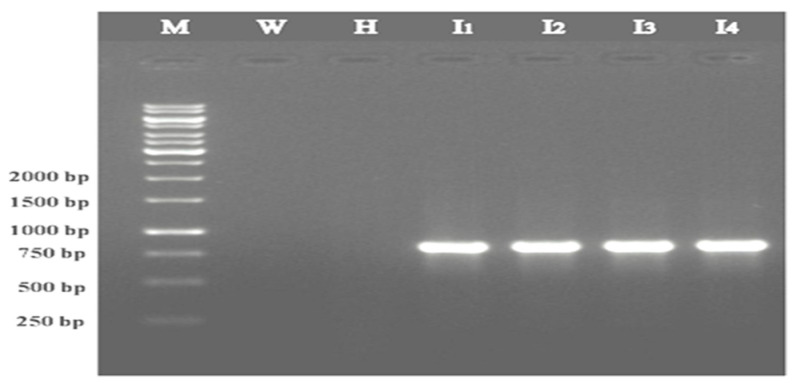
Agarose gel showing PCR amplification of the CP gene of ToLCNDV-Rg infecting the ridge gourd. Lane M: 1 kb molecular marker; Lane W: Water control; Lane H: Healthy sample; Lane I_1_, I_2_, I_3_, and I_4_: Infected ridge gourd samples.

The ToLCNDV-Rg-CP gene was amplified by PCR (Figure 1) from the infected ridge gourd leaf sample. The amplified PCR product was purified and cloned into pBluescriptII KS+ (Figure S1a) and sub-cloned in pET22-NH (Figure S1b). Further recombinant clones were confirmed by restriction digestion with *BamH*1/*Hind*III and *Nde*I/*Hind*III enzymes, respectively. The identity of the clone was further confirmed by sequencing the CP gene.

**Figure 2:**
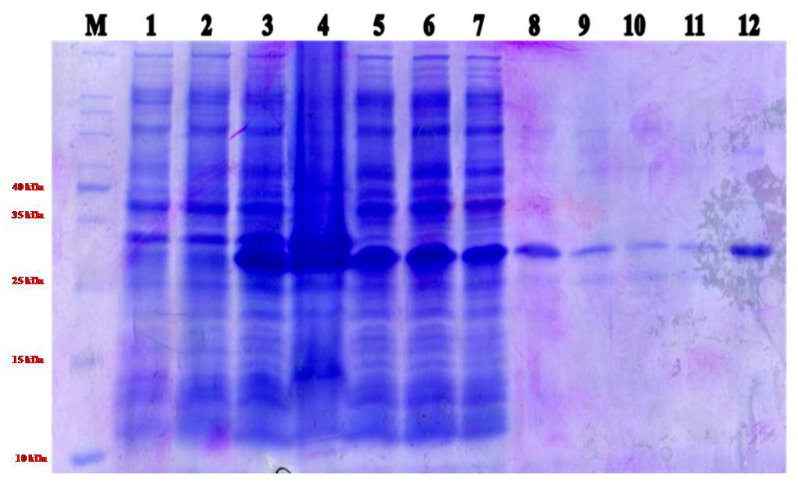
SDS-PAGE (14%) showing expression of recombinant ToLCNDV-Rg-CP in *Escherichia coli* under 0.3 mM IPTG and purification of recombinant ToLCNDV-Rg- CP using 0.5 % triton-X 100. Lanes M: Protein Marker; Lane 1: *E. coli* BL-21 cells; Lane 2: Uninduced, Lane 3: Whole cell lysate, Lane 4: Pellet, Lane 5: Supernatant, Lane 6: Flow through; Lane 7: Beads before wash; Lane 8: Beads after wash; Lane 9–11: Eluted protein; Lane 12: Concentrated protein.

Of the different concentrations of IPTG (0.05 mM to 0.5 mM) and temperature conditions (20 °C to 37 °C) tested, 0.3 mM IPTG at 20 °C was recorded as the highest expression of ToLCNDV-Rg-CP of approximately 30.5 kDa in size (Figure S2). Most of the recombinant ToLCNDV-Rg-CP was observed in the pellet fraction of the sonicated cell lysate but was suspended in a buffer containing 0.5% Triton X-100, suggesting that the protein was associated with membrane fraction. The protein was purified using Ni-NTA-agarose affinity chromatography (Figure 2). SDS-PAGE and WB analyses revealed an approximate molecular weight of 30.5 kDa for the NH-ToLCNDV-Rg-CP recombinant protein. Immunization of rabbits by single subcutaneous and four intramuscular injections with purified NH-ToLCNDV-Rg-CP successfully produced antibodies (ToLCNDV-Rg-CP-PAb) against the viral protein. The recombinant protein was found to be highly immunogenic, and the booster doses increased the titre.

### 2.2. Sensitivity of ToLCNDV-Rg-CP-PAb to Recombinant Protein

The antiserum detected the recombinant protein up to a dilution of 1:40,000 (Table S1). The mean OD values of DAC-ELISA with 1:100 to 1:40,000 dilutions of ToLCNDV-Rg-CP-PAb were found to be two times higher than the mean values of the buffer control (Table S1). Based on the above results, the ToLCNDV-Rg-CP-PAbs can be diluted up to 1:40,000 for detecting the ToLCNDV-Rg-CP. The antibody was sensitive to detect the recombinant antigen when the wells were coated with a solution of 0.375 µg of the antigen (Table S2). The antigen was also detected using similar antibody dilutions by WB. Similar experiments in DAC-ELISA and DIBA, however, detected ToLCNDV-Rg in antibody dilutions of up to only 1:20,000 (Table S3). No significant reaction was observed with extracts from healthy plant tissues, suggesting that the antibody is specific to the CP.

### 2.3. DIBA

Both purified recombinant ToLCNDV-Rg-CP and ToLCNDV-Rg-infected samples showed dark spots on the nitrocellulose membrane in DIBA but not from healthy samples and buffer control (Figure S3). The intensity of the colour development showed the degree of sensitivity of detection for ToLCNDV. The optimal ToLCNDV-Rg-CP-PAb titre for the detection of ToLCNDV-Rg by DIBA was observed to be 1:20,000.

### 2.4. WB

**Figure 3:**
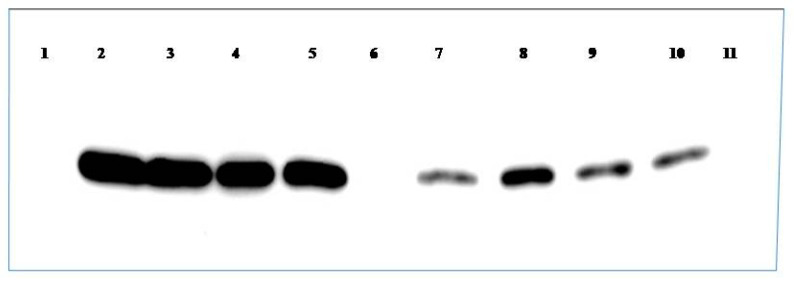
Western blot analysis of purified ToLCNDV-Rg-CP and ToLCNDV-Rg isolate infected plant sample with the ToLCNDV-Rg-CP-PAbs. Lane 1: Blank *E.coli* BL21 strain; Lanes 2–5: Purified ToLCNDV-Rg-CP expressed in *E. coli*.; Lane 6: Crude plant extract from healthy ridge gourd; Lanes 7–10: Crude plant extract from ToLCNDV-Rg infected ridge gourd; Lane 11: pET22-NH+ *E.coli* BL21 strain.

WB revealed 30.5 kDa CP protein from ToLCNDV-Rg-infected leaf extracts as well as the purified recombinant ToLCNDV-Rg-CP but not from healthy ridge gourd leaves (Figure 3).

### 2.5. Immunocapture Assays (IC-PCR and IC-LAMP)

**Figure 4:**
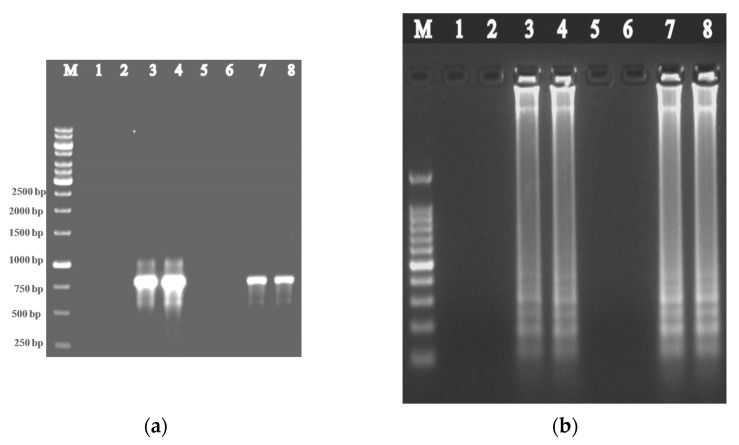
Agarose gel showing the amplification of ToLCNDV-Rg-CP gene from non-immunocaptured and immunocaptured plant samples using ToLCNDV- CP specific primers through (**a**) IC-PCR and (**b**) IC-LAMP assays. Lane M: 1 Kb DNA marker; Lanes 1 and 5: Water control; Lanes 2 and 6: Healthy samples; Lanes 3 and 4: Infected ridge gourd samples; Lanes 7 and 8: Infected immunocaptured ridge gourd samples.

The IC-PCR and IC-LAMP assays were performed to capture the virus particles directly from the supernatant obtained from infected plant sap. The resulting IC-PCR amplifications produced a fragment of the size of 771 nt from ToLCNDV-Rg-infected ridge gourd samples but not from healthy samples and water control (Figure 4a). In the case of IC-LAMP assay, ladder-like DNA amplifications of different sizes were observed from ToLCNDV-Rg-infected ridge gourd samples, whereas no ladder-like DNA amplification products were seen in the healthy samples and water control (Figure 4b).

### 2.6. Determining the Efficacy of ToLCNDV-Rg-CP-PAbs in Immmunocapture Assays

**Figure 5:**
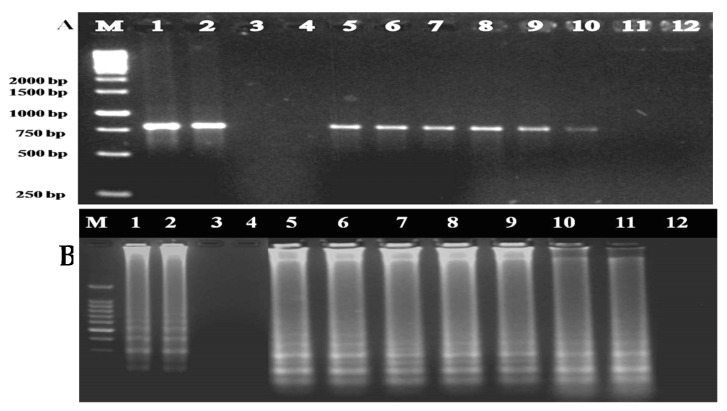
Comparative analysis of the sensitivity of IC-PCR and IC-LAMP using serial dilutions of ToLCNDV-Rg-CP-PAb (**A**) IC-PCR, (**B**) IC-LAMP. Lanes 1 and 2: Positive samples; Lane 3: Water control; Lane 4: Healthy control; Lane 5: 1:2500 dilutions of the antibody; Lane 6: 1:5000; Lane 7: 1:10,000; Lane 8: 1:20,000; Lane 9: 1:40,000; Lane 10: 1: 80,000; Lane 11: 1:1,000,000; Lane 12: 1:2,000,000.

For determining the comparative sensitivities of immunocapture assays, the supernatant obtained from crude leaf extract of the ridge gourd plant infected with ToLCNDV-Rg isolate was used to capture the virus using serially diluted ToLCNDV-Rg-CP-PAbs, and the captured virus particles were used as a source of template in PCR and LAMP assays. The results demonstrated that the virus could be detected using antibody dilutions up to 1:80,000 and 1:100,000 in IC-PCR (Figure 5A) and IC-LAMP (Figure 5B) assays, respectively, indicating IC-LAMP was more sensitive than IC-PCR assay.

### 2.7. Broad-Spectrum Detection of Begomoviruses Using ToLCNDV-Rg-CP-PAb

**Figure 6:**
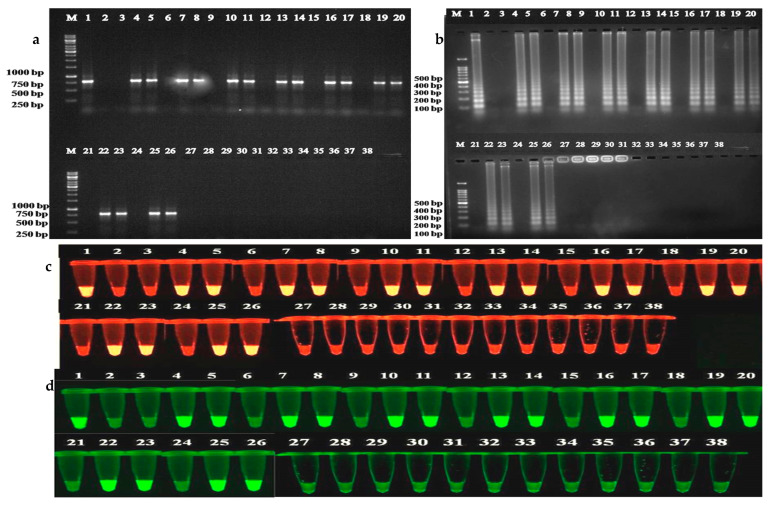
Immunocapture assays for samples from various host plants suspected to be infected with begomoviruses and their visualization on agarose gel using dyes (**a**) IC-PCR, (**b**) IC-LAMP, (**c**) EtBr, and (**d**) VeriPCR dyes. Lane M: 1 kb DNA marker; Lane 1: Positive control (Infected ridge gourd); Lane 2: Negative control (Buffer); Lane 3: Healthy ridge gourd; Lane 4, 5: Infected ridge gourd; Lane 6: Healthy watermelon; Lane 7, 8: Infected watermelon; Lane 9: Healthy cucumber; Lane 10, 11: Infected cucumber; Lane 12: Healthy pumpkin; Lane 13, 14: Infected pumpkin; Lane 15: Healthy bitter gourd; Lane 16, 17: Infected bitter gourd; Lane 18: Healthy bottle gourd; Lane 19, 20: Infected bottle gourd; Lane 21: Healthy sponge gourd; Lane 22, 23: Infected sponge gourd; Lane 24: Healthy tomato; Lane 25, 26: Infected tomato; Lane 27: Healthy chilli; Lane 28, 29: Infected chilli; Lane 30: Healthy capsicum; Lane 31, 32: Infected capsicum; Lane 33: Healthy cowpea; Lane 34, 35: Infected cowpea; Lane 36: Healthy French bean; Lane 37, 38: Infected French bean.

To investigate the cross-reactivity of the antibodies, leaf samples from various crops infected with five begomoviruses (Table 1) were tested. Begomoviruses were in the samples detected by PCR using core CP primers with the expected amplicon size of 575 nt (Figure S4). Similarly, 771 nt size amplicons were observed in begomovirus-infected bitter gourd, bottle gourd, cucumber, pumpkin, ridge gourd, snake gourd, and watermelon samples, but not in chilli, capsicum, cowpea, and French bean and healthy samples using ToLCNDV-CP specific primers (data not shown). LAMP assay produced similar results as PCR, on agarose gel (Figure S5a), with EtBr (Figure S5b) and VeriPCR (Figure S5c) dyes. Similar results were obtained in IC-PCR (Figure 6a) and IC-LAMP assays (Figure 6b) with EtBr (Figure 6c) and VeriPCR (Figure 6d) dyes from all the infected samples except the four crops: chilli, capsicum, cowpea, and French bean. DAC-ELISA results also showed positive results from all the infected samples except the same four crops. The OD values obtained ranged from 0.84 to 1.40 in infected samples compared to 0.23 to 0.40 in healthy samples (Table S4).

The optimized DAC-ELISA was further validated by testing 20 RgYMD-affected field samples from southern India. All samples showed positive reactions with the OD values from the ELISA test ranging from 0.721 to 1.421 (Table S5).

### 2.8. Sequence Analysis of ToLCNDV-Rg Isolate CP Gene with the Other Begomoviruses

The complete DNA-A sequence analysis indicated that watermelon was infected by ToLCPalV, pumpkin (yellow mosaic) by SLCCNV and cucumber, bitter gourd, bottle gourd, snake gourd, and pumpkin (leaf curl) by ToLCNDV, chilli and capsicum by ChiLCV, cowpea and French bean by MYMIV. Comparative analysis of ToLCNDV-Rg-CP gene nucleotide (nt) sequences associated with ridge gourd disease with those of other begomoviruses reported previously showed high nt identity with ToLCNDV (88.4–98.5%), ToLCPalV (80.9–93.1%), SLCCNV (83.1–92.3%) than to ChiLCV (70.1–78.2%) and MYMIV (61.1–62.9%). ToLCNDV-Rg-CP amino acid (aa) sequence analysis also revealed a very high identity with ToLCNDV (91.7–98.8%), ToLCPalV (86.7–92.5%), SLCCNV (90.0–96.4%) as compared to ChiLCV (74.6–78.9%) and MYMIV (74.6–83.8%).

## 3. Discussion

Viruses belonging to the genus *Begomovirus* in the family *Geminiviridae* are a major cause of severe yield loss and affect the quality and quantity of produce in several crops [6,7]. Diagnostic methods are necessary for regular surveillance, quarantine, and eradication programs to keep the crops safe from virus infections to prevent crop losses incurred. Availability and cost-effective detection methods for begomoviruses infecting economically important crops and in their vector whitefly are also essential for epidemiological studies and disease management. Early detection of infected plants is important to prevent the further spread of viruses within a crop, thus reducing yield losses. The ridge gourd is an important vegetable in India, which is severely affected by RgYMD caused by ToLCNDV-Rg [3].

Obtaining pure virus particles from infected plants for antibody production is time-consuming, due to the problems of contamination by host proteins, inhibitory substances, and/or low virus yields [24] and many times might be outright impossible because of mixed infection of begomoviruses [25]. Therefore, in this study, recombinant DNA technology was used for expressing the conserved CP coding region of ToLCNDV-Rg to generate polyclonal antibodies. Production of recombinant CP eliminated the need for the maintenance of plants as well as the time-consuming virus purification process, which made it possible to standardize the process of quick purification of the antigen for producing PAbs [26]. In addition, the recombinant clone can be stored indefinitely and used for antigen preparation to ensure the continuous supply of purified CP for the long-term production of PAbs. High-level expression of the recombinant protein of ToLCNDV-Rg-CP with His-tag was obtained with 0.3 mM IPTG in membrane-bound fraction. Extraction of the recombinant protein in native soluble form with the aid of a small amount of detergent facilitated its easy purification by Ni-NTA-agarose affinity chromatography. Since the N-terminal His tag was not expected to have significant immunogenic properties [27], the affinity-purified ToLCNDV was successfully used to produce diagnostic PAbs.

The specificity of ToLCNDV-Rg-CP-PAb to ToLCNDV-Rg-CP was evaluated by DIBA, ELISA, and WB assays. The antibody reacted positively with extracts from ToLCNDV-Rg infected ridge gourd leaf samples and purified protein but not from healthy or buffer controls. The DIBA results also showed strong signals for ToLCNDV, ToLCPalV, and SLCCNV-infected samples from other crop plants. No positive signals were, however, detected with the extracts of ChiLCV and MYMIV-infected samples and healthy plant samples. DIBA is a simple, sensitive, economical, and field-applicable technique as the results can be viewed visually without any instrument [28,29].

The two optimized immunocapture assays (IC-PCR and IC-LAMP) showed that the ToLCNDV-Rg-CP-PAb can be used at 1:80,000 dilution for capturing the virus for use in these assays. They also eliminate the need for the isolation of nucleic acids in the standard PCR. IC-LAMP was found to be more sensitive than IC-PCR as it detected the viruses captured at 1:100,000 antibody dilutions compared to 1:80,000 by IC-PCR. Moreover, IC-LAMP does not require sophisticated equipment and hazardous chemicals such as ethidium bromide. Similarly, Almasi et al. [23] optimized the IC-LAMP assay for the detection of TYLCV in tomatoes. WB, ELISA, and IC–PCR are expensive methods that can be used only in well-equipped laboratories with trained personnel, while the IC–LAMP can be performed using just a water bath or temperature block [23,30]. IC-LAMP was therefore adapted for on-site detection of ToLCNDV using antibodies produced in this study, and the detection time was shortened to 50 min as compared to IC-PCR.

Begomoviruses often occur in mixed infections in fields [25]. Furthermore, depending upon the host plant’s species and/or its genetics, different variants/isolates of begomoviruses can be dominant in a given infected plant [31,32]. Antibodies generated in this study strongly reacted with the extracts from different plants infected with ToLCNDV-Rg and other related begomoviruses endemic in the region. Taken together, antibodies obtained using recombinants CP of ToLCNDV not only provide broad sensitivity against naturally occurring ToLCNDV variants/isolates but also against other begomoviruses such as ToLCNDV, ToLCPalV, and SLCCNV. Cross sensitivity against related begomoviruses might be the result of shared CP epitome profiles [33]. Nevertheless, PAbs generated in this study can be used for detecting two other viruses: ToLCPalV and SLCCNV, but not ChiLCV and MYMIV, which are antigenically unrelated. Other molecular assays such as PCR, real-time PCR, and sequencing have been used for diagnosing plant viruses, however, they are costly, time-consuming, and require sophisticated equipment. We recommend the IC-LAMP method for diagnosing ToLCNDV-Rg and related viruses as it is equally sensitive but quick and more affordable.

## 4. Materials and Methods

### 4.1. Virus Samples and DNA Isolation

Ridge gourd plants infected with ToLCNDV and showing RgYMD were maintained in an insect-proof net house at the College of Horticulture, Bengaluru, University of Horticultural Sciences, Bagalkot, India [3]. Leaf samples from different crop plants such as watermelon (*Citrullus lanatus* (Thunb.) Mansf.), cucumber (*Cucumis sativus* L.), pumpkin (*Cucurbita moschata* Duchesne), bitter gourd (*Momordica charantia* L.), bottle gourd (*Lagenaria siceraria* (Molina) Standl.), snake gourd (*Trichosanthes cucumerina* L.), chilli (*Capsicum annuum* L.), capsicum (*Capsicum annuum* L.), cowpea (*Vigna unguiculata* (L.) Walp), and French bean (*Phaseolus vulgaris* L) showing typical symptoms of begomovirus infection such as leaf curling, leaf yellowing, yellow vein mosaic, distorted and stunted growth of the plants were collected from farmer fields in and around Bengaluru, Karnataka State, India for validating the diagnostic use of PAbs produced against the ToLCNDV-Rg-CP. Total nucleic acids were isolated from these samples by the CTAB method [34,35] prior to use in diagnostic tests.

### 4.2. Construction of ToLCNDV-Rg-CP Plasmid, Expression, and Purification of Protein

ToLCNDV-CP was amplified from DNA extracted from RgYMD leaf samples using CP gene-specific primers (Table S6). The amplified CP gene fragment was purified and cloned into a cloning vector (pBluescriptII KS+). The insert from a positive clone was transferred into a modified pET22 bacterial expression vector (pET22-NH) between *Nde*I and *Hind*III sites to generate a fusion construct (pET22-NH-CP) for expressing CP in fusion with a hexa histidine tag at the N terminus (NH) [36]. *Escherichia coli* BL21 (*DE3*) was transformed with plasmid pET22-NH-CP DNA, and recombinant clones were selected on LB plates containing 100 µg/mL of ampicillin. The bacterial culture (1000 mL) was incubated overnight in the presence of 0.05 mM IPTG at 20 °C in a shaker at 120 rpm to induce the protein expression. The bacterial suspension was centrifuged at 6000 rpm for 10 min at 4 °C. The pellet was washed by vertexing with a 2 mL lysis/binding buffer (10 mM Tris HCl, 150 mM NaCl, 0.5 % Triton X-100; pH adjusted to 7.4 with NaOH) obtained from every 200 mL cell suspension. Tubes were further centrifuged at 12,000 rpm for 10 min, and the pellet (~100 mL suspension) obtained was re-suspended in a 10 mL lysis buffer. Of this, 20 µL (whole cell lysate) was loaded onto sodium dodecyl sulfate-polyacrylamide gel electrophoresis (SDS-PAGE), and the remaining culture was used for protein purification. Cells were disrupted by sonication with 20–25 pulses at 30% power using a sonicator (Sonics-Vitra cell, New York, USA). Five ml of lysis buffer was added to the cell lysate, then mixed gently and incubated on ice for 30 min. The cell lysate was centrifuged at 17,000 rpm for 45 min at 4 ºC. The supernatant was transferred into the nickel-nitrilotriacetic acid (Ni-NTA)-agarose column containing 1.0 mL of beads (The QIAexpressionist, Qiagen, New Delhi, India) for protein binding. A small amount of pellet fraction, 20 µL of supernatant, and flow through from the column were collected. The columns were placed on a rotator for 90 min for the binding of proteins to the Ni-NTA-agarose beads. The protein-bound beads were washed with 10 mL wash buffer-I (10 mM Tris-HCl, 150 mM NaCl, and 40 mM imidazole, pH 7.4) 5 times and wash buffer-II (10 mM Tris-HCl, 150 mM NaCl, pH 7.4) 5 times. Around 10 µL of beads bound with ToLCNDV-Rg-CP were collected before and after washing with wash buffer. About 200 µL of elution buffer (10 mM Tris-HCl, 100 mM NaCl, 500 mM Imidazole, 10% Glycerol) was added to the column after the closing of the outlet of the column. The column was left for 1 h, and the flow through was collected. This elution step was repeated three times. The purified protein from three elutions (600 µL) was mixed. Fifty percent glycerol was added to obtain a final concentration of 10% glycerol, then stored at −80 °C and subjected to dialysis when PAbs were required. ToLCNDV-Rg-CP recombinant protein was subjected to step dialysis in 250 mL of dialysis buffer-I (10 mM Tris-HCl, 100 mM NaCl, 100 mM Imidazole, 5 % Glycerol) and incubated for 2 h on a magnetic stirrer at room temperature. Dialysis bags containing purified protein was transferred to dialysis buffer-II (10 mM Tris-HCL, 100 mM NaCl, 5% Glycerol) (second buffer) and incubated for 2 h followed by replacement with a 1/4th, 1/2th, 3/4th volume of change buffer (Second buffer) and incubated for 2 h each. Finally, the dialysis bag was kept in a fresh change buffer and incubated overnight at 4 °C on a magnetic stirrer (Remi, Mumbai, India). The expression level was optimized in harvested cells by different time course induction with IPTG. The expression level was also optimized for different temperatures and IPTG concentrations. The concentration of purified protein was estimated by the Bradford method [37].

### 4.3. Protein Separation by Sodium Dodecyl Sulfate Polyacrylamide Gel Electrophoresis (SDS-PAGE)

SDS-PAGE was carried out according to the procedure described by Laemmli [38]. Twenty microlitre of the sample or purified protein was mixed with 20 of Laemmli buffer, heated for 10 min at 95 °C, and loaded on a 14% SDS PAGE gel. A pre-stained protein molecular weight marker (Cat No. SM0441, Fermentas, Eschenstr, Germany) (5 µL) was used for comparison of the molecular weight of the recombinant protein. Electrophoresis was carried out at 70 V until the dye front reached the resolving gel and then at 90 V for 4 h.

### 4.4. Sequence Analysis of ToLCNDV-Rg-CP

The plasmid DNA containing ToLCNDV-Rg CP gene insert was sequenced at Medauxin Pvt. Ltd., Bengaluru, India, with M13 forward and reverse primers using ABI Prism Big Dye Terminator Version 3.1 Cycle Sequencing Kit (Applied Biosystems, Foster City, CA, USA) according to the manufacturer’s instructions. The nucleotide (nt) and the deduced amino acid (aa) sequences of the ToLCNDV-Rg-CP gene were aligned and compared with other isolates of ToLCNDV available in the NCBI GenBank database.

### 4.5. Immunization of Rabbit and Serum Collection

Production of antiserum against recombinant ToLCNDV-Rg-CP was carried out at Genei Laboratories Pvt. Ltd., Bengaluru, India. ToLCNDV-Rg-CP polyclonal antibodies (ToLCNDV-Rg-CP-Pab) were raised in a six-month-old female New Zealand white rabbit (*Oryctolagus cuniculus*) by injecting purified ToLCNDV-Rg-CP protein. One milligram of purified ToLCNDV-Rg-CP (500 µL) protein was emulsified with an equal volume of Freund’s complete adjuvant (GeNei™, Cat. No. 105474, Genei Laboratories, Bengaluru, India) and injected at five sites, subcutaneously in the back region using a 2 mL syringe. Four subsequent booster doses of approximately 0.5 mg (500 µL) of ToLCNDV-Rg-CP protein emulsified with Freund’s incomplete adjuvant (GeNei™, Cat. No. 105475, Genei Laboratories, Bengaluru, India) were injected at weekly intervals into two sites intramuscularly in the thigh region. Pre-immunization test bleeding was done just before the administration of the first dose by collecting 1 mL of blood from the outer vein of the ear. Test bleeding was done one week after the third immunization to check the antibody titre. One week after the last injection, the rabbit was bled five times at two-day intervals. 5–10 mL blood was drawn each time from the leg vein into falcon tubes. Immediately after each bleeding, the tubes were kept in a slanted position for 1 h to facilitate serum separation. The tubes were then kept at 4 °C overnight in a slanted position in a refrigerator. The tubes were centrifuged at 3000 rpm for 10 min. The supernatant was collected into a fresh tube and further centrifuged at 12,000 rpm for 10 min. Clear supernatant (crude serum) was transferred into a new tube. The antiserum obtained was mixed with 50 % glycerol to obtain a final concentration of 10 % glycerol and stored at −80 °C.

### 4.6. Antibody Tests

The immunodiagnostic efficacy of the ToLCNDV-Rg-CP-PAb was evaluated by DAC-ELISA, DIBA, WB, IC-PCR, and IC-LAMP assays.

#### 4.6.1. Direct Antigen Coating Enzyme-Linked Immuno Sorbent Assay

To evaluate the efficacy of ToLCNDV-Rg-CP-PAb, DAC-ELISA was carried out using the antiserum according to the method developed by Clark and Bar-Joseph [39]. Proteins from ToLCNDV-Rg infected and healthy ridge gourd plants were extracted using the extraction buffer (6.06 g Tris, 4.68 g sodium sulfite were dissolved in distilled water to 1000 mL, pH 8.5, stored at 4 °C), and an equal amount (100 μL) of the extract was added to the wells in triplicate in a microtiter plate and incubated at 37 °C for 2 h. The wells were washed thrice using a wash buffer (PBS) containing 0.05% (*w*/*v*) Tween-20, followed by blocking by incubating with a solution of 5% bovine serum albumin (BSA) for one hour at 37 °C and washing thrice as described in the previous section. The serially diluted (1:100 to 1:40,000) antiserum (ToLCNDV-Rg-CP-PAb) was added to triplicate wells coated with antigen and incubated at 37 °C for 2 h and washed thrice. Secondary anti-rabbit goat IgG conjugated with alkaline phosphatase (Sigma-Aldrich, India) (1:15,000 dilution) was added and incubated at 37 °C for 2 h. After the final washing, p-nitrophenol phosphate (pNPP) (0.5 mg/mL; Sigma Aldrich, St. Louis, MO, USA) substrate prepared in 10% diethanolamine buffer (pH 9.8) was added to the wells. The absorbance value of each well was measured at 405 nm using a microtiter plate reader (BIO-TEK Instruments Inc., Winooski, VT, USA). Wells showing mean absorbance values twice as high as the healthy controls were considered positive. To determine the minimum quantity of viral antigen required for ELISA detection, purified recombinant ToLCNDV-Rg-CP at different concentrations (1.8, 3.75, 7.5, 15, 30, 60, 125, 250, 500 µg/mL) was used. The optimized DAC-ELISA assay was used to demonstrate RgYMD in 20 ridge gourd field samples collected from plants affected by the disease from different locations in Southern India.

#### 4.6.2. Dot Immuno Binding Assay

A piece of the nitrocellulose membrane of the desired size (Type-SCN, 0.45) was cut and soaked in TBS for 10 min and then air dried for 15 min on filter paper. The supernatant obtained from crude sap was extracted from the ToLCNDV-Rg-infected and healthy ridge gourd leaf tissues prepared separately by grinding 0.2 g of leaf tissues in 10 mL of antigen extraction buffer, grounded tissues were then mixed with an equal amount of chloroform and centrifuged at 10,000 rpm for 5 min, the clear supernatant (5–10 µL) from each sample was manually spotted onto the membrane [40]. The membrane was air dried for 15 min and incubated with blocking solution for one hour with gentle shaking followed by rinsing in a washing buffer (TBS-T) for 10 min. To test the dilution endpoint, the membranes were incubated with equal volumes of serially diluted (1:100 to 1:20,000) ToLCNDV-Rg-CP-PAb for one hour, followed by rinsing the membrane three times with a wash buffer (TBS-T). Then the membrane was incubated with goat anti-rabbit IgG-Alkaline phosphatase (ALP) conjugate (Sigma Aldrich, St. Louis, USA) (1:15,000 dilution) for one hour with gentle shaking on a rocker and rinsed with a wash buffer. BCIP/NBT (Sigma Aldrich, St. Louis, USA) solution (1 mL) was spread over the membrane over a plastic sheet and kept for 30 min in the dark for color development. Membranes were then rinsed thrice with a fixing solution for 10 min, air dried, and were photographed using a digital camera.

#### 4.6.3. Western Blotting

WB was carried out as per the standard procedure provided by Towbin et al. [41]; leaf tissues of 0.2 g from healthy and infected ridge gourd plants were homogenized in 5 volumes of sample extraction buffer (6.06 g Tris, 4.68 g sodium sulfite dissolved in distilled water to 1000 mL, pH 8.5, stored at 4 °C). Extracts were heated for 10 min at 100 °C after adding the Laemmli buffer. Proteins in the crude extracts from ToLCNDV-Rg infected and healthy ridge gourd leaf samples were analyzed along with 1 µg of expressed ToLCNDV-Rg-CP by SDS-PAGE on 14% gel. Separated proteins were electro-blotted onto a Millipore polyvinylidene difluoride (PVDF) membrane (Millipore, Burlington, USA). Membranes were blocked with a 5% skimmed milk solution in TBS for 3 h at room temperature to reduce the nonspecific binding of the antibody. The membrane was washed 3 times for 10 min using TBS-T on a rocker. Then the membrane was incubated with primary antibody solution (ToLCNDV-Rg-CP-PAb (1:5000 dilution) for 2 h with gentle shaking and washed with TBS-T buffer 3 times on a rocker. The membrane was then incubated with anti-rabbit horse radish peroxidase (HRP) conjugated with a secondary antibody in TBS-T (1:5000 dilution) for 2 h with gentle shaking and was washed 3 times with TBS-T buffer. The blot was developed using an enhanced chemiluminescence (ECL) kit (Immunobilon™ Western, Millipore, Burlington, USA) as per protocol provided by the manufacturer.

#### 4.6.4. Immunocapture Polymerase Chain Reaction and Immunocapture Loop Mediated Isothermal Amplification

Approximately 150 µL of ToLCNDV-Rg-CP-PAbs, diluted (1:100 to 1:200,000) with coating buffer, was added to polypropylene microcentrifuge tubes. The tubes were incubated at 37 °C for 2 h, washed 3 times with PBS-T, and air dried. Crude leaf extracts of ToLCNDV-Rg infected and healthy ridge gourd plants were prepared separately by homogenizing 100 mg of the leaf sample in a 1 mL sample extraction buffer. Extracts were centrifuged at 10,000 rpm for 5 min, and the supernatant was transferred to new tubes. To the ToLCNDV-Rg-CP-PAb-coated tubes, the supernatant (150 μL) was added and incubated at 37 °C for 2 h. The tubes were washed 3 times with PBS-T and air-dried. Finally, 10 μL of nuclease-free water was added and incubated at 95 °C for 5 min, followed by incubation on ice. Approximately 5 μL of the nuclease-free water containing the viral DNA template was used for IC-PCR and IC-LAMP assays. The IC-PCR was carried out using CP gene-specific primers (Table S6), and the IC-LAMP assay was carried out as described by Naganur et al. [11]. VeriPCR and EtBr dyes were used for the visual detection of positive reactions [11].

### 4.7. Broad-Specificity Analysis of the ToLCNDV-Rg-CP-PAb for the Detection of Begomoviruses Infecting Other Crops

#### 4.7.1. Virus Infected Samples and DNA Isolation

Leaf samples from different crops such as watermelon, cucumber, pumpkin, bitter gourd, bottle gourd, snake gourd, chilli, capsicum, cowpea, and French bean showing typical begomovirus symptoms as well as samples from healthy plants were collected from fields in and around Bengaluru, India during the months of March–April in 2017 for determining the broad reactivity of the antibody against begomoviruses infecting a variety of vegetable crop plants. Total genomic DNA was extracted from the healthy and infected leaf samples using the CTAB method and stored at −20 °C for further analysis.

#### 4.7.2. Detection of Begomoviruses in Different Crop Plants

Degenerate primers [42] (Table S6) were used to amplify the core-CP region of begomovirus to detect the presence of the begomoviruses in the infected samples. CP gene-specific primers (Table S6) were used to detect the presence of ToLCNDV in the infected samples. All the samples were subjected to DAC-ELISA, DIBA, IC-PCR, and IC-LAMP assays to check the broad specificity as well as the sensitivity of the ToLCNDV-Rg-CP-PAb produced in the current study.

#### 4.7.3. Sequence Analysis of Begomoviruses Associated with Other Crops

The PCR amplification of complete DNA-A from different crops infected with begomoviruses was carried out according to Venkataravanappa et al. [43]. The amplified products were purified, cloned, and sequenced by primer walking at Medauxin Pvt. Ltd., Bengaluru, India. The sequences of full-length DNA-A were obtained and subjected to BLAST analysis to identify the presence of specific begomoviruses associated with crop plants [44]. Analysis of nucleotide sequences of DNA-A and CP gene and amino acid sequence of CP for present isolates with sequences obtained from the NCBI database (Table S7) was done using the Sequence Demarcation Tool (SDT) version 1.2 [45].

## Figures and Tables

**Table 1:** Begomoviruses associated with different hosts were used for broad spectrum detection assays.

Sl No	Begomovirus	Hosts
1	Tomato leaf curl New Delhi virus (ToLCNDV)	Bitter gourd, bottle gourd, cucumber, ridge gourd, snake gourd, and pumpkin
2	Tomato leaf curl Palampur virus (ToLCPalV)	Watermelon
3	Squash leaf curl China virus (SLCCNV)	Pumpkin
4	Chilli leaf curl virus (ChiLCV)	Chilli and capsicum
5	Mungbean yellow mosaic India virus (MYMIV)	Cowpea and French bean

## Supplementary Materials

The following supporting information can be downloaded at: https://www.mdpi.com/article/10.3390/plants12030490/s1, Table S1. Detection of ToLCNDV through DAC-ELISA with different dilutions of the polyclonal antibody ToLCNDV-Rg-CP-Pab; Table S2. Sensitivity of ToLCNDV-Rg-CP-Pab with different concentrations of antigen (ToLCNDV-Rg-CP) determined by DAC-ELISA technique; Table S3. Detection of ToLCNDV through DAC-ELISA with different dilutions of ToLCNDV-Rg-CP-Pab; Table S4. Enzyme-linked immuno sorbent assay showing the reaction of samples from different hosts using ToLCNDV-Rg-CP-Pab; Table S5. Validation of DAC-ELISA by detection of ToLCNDV in plant samplescollected from various fields in South India; Table S6. Primers used in the present study for amplification of ToLCNDV coat protein gene; Table S7. GenBank accession numbers of selected begomovirus sequences from database used in this study for analysis of DNA-A component and coat protein; Figure S1. Agarose gel photograph showing restriction digestion of recombinant pBluescript IIKS(+)-ToLCNDV-Rg isolate-CP with *BamH*I and *Hind*III restriction endonucleases (a) and Restriction digestion of recombinant pET22-NH-ToLCNDV-Rg-CP with *Nde*I and *Hind*III restriction endonucleases (b); Figure S2. Expression of ToLCNDV-Rg-CP in *E. coli* at different concentrations of IPTG and incubation temperatures; Figure S3. Dot immuno binding assay (DIBA) with different dilutions of ToLCNDV-Rg-CP-Pab tested with ToLCNDV-Rg-CP; Figure S4. Agarose gel showing PCR amplification of core CP of begomoviruses from various infected host plants; Figure S5. LAMP amplification of begomoviruses from various crops suspected to be infected with begomovirus and its visualization on (a) 2 % agarose gel (b), EtBr and (c) VeriPCR dyes.
