# Supplementary material for: Detecting Tomato Leaf Curl New Delhi Virus Causing Ridge Gourd Yellow Mosaic Disease, and Other Begomoviruses by Antibody-Based Methods
Source: Plants (Basel). 2023 Jan 20;12(3):490. doi: 10.3390/plants12030490 (PMC9919216; doi:10.3390/plants12030490)
Supplement: Supplementary file 1 [file plants-12-00490-s001.zip › plants-2057274-supplementary.pdf]

Table S1. Detection of ToLCNDV through DAC-ELISA with different dilutions of the polyclonal antibody ToLCNDV-Rg-CP-Pab

Sl. No	ToLCNDV-CP-Rg-Pab dilution	OD value at 405 nm (Mean of 3 replicates)	
		Buffer ±SEM	ToLCNDV-CP-Rg ±SEM
1	1:100	0.53 ±0.0243	1.03 ± 0.0472
2	1:500	0.42 ±0.0147	0.83 ± 0.0291
3	1:1000	0.36 ±0.0091	0.77 ± 0.0194
4	1:2000	0.30 ±0.0046	0.75 ± 0.0115
5	1:4000	0.28 ±0.0113	0.68 ± 0.0275
6	1:8000	0.25 ±0.0115	0.65 ± 0.0298
7	1:10000	0.24 ±0.0037	0.64 ± 0.0098
8	1:20000	0.22 ±0.0038	0.62 ± 0.0107
9	1:40000	0.20 ±0.0070	0.55 ± 0.0193
10	1:80000	0.20 ±0.0070	0.30 ± 0.0105
11	1:100000	0.18 ±0.0063	0.25 ± 0.0088
12	1:200000	0.15 ±0.0053	0.15 ± 0.0053
13	Conjugate control	0.04 ±0.0014	0.06 ± 0.0021
14	Substrate control	0.04 ±0.0014	0.05 ± 0.0018

Table S2. Sensitivity of ToLCNDV-Rg-CP-Pab with different concentrations of antigen (ToLCNDV-Rg-CP) determined by DAC-ELISA technique

Antigen concentration (µg/ml)	OD value at 405 nm (Mean of 3 replicates)	
	Buffer	ToLCNDV-CP-Rg
500	0.16 ± 0.0073	1.34 ± 0.0614
250	0.15 ± 0.0053	0.95 ± 0.0255
125	0.13 ± 0.0033	0.75 ± 0.0075
60	0.13 ± 0.0020	0.71 ± 0.0222
30	0.12 ± 0.0044	0.30 ± 0.0121
15	0.12± 0.0050	0.25 ± 0.0115
7.5	0.11 ± 0.0046	0.22 ± 0.0094
3.75	0.10 ± 0.0046	0.20 ± 0.0092
1.80	0.10 ± 0.0046	0.10 ± 0.0046

Table S3. Detection of ToLCNDV through DAC-ELISA with different dilutions of ToLCNDV-Rg-CP-Pab

Antibody dilution	OD value at 405 nm (Mean of 3 replicates)		
	Buffer control	Healthy ridge gourd	Infected ridge gourd
1:100	0.60 ± 0.0274	0.76 ± 0.0348	2.10 ± 0.0962
1:500	0.51 ± 0.0233	0.63 ± 0.0280	1.51 ± 0.0691
1:1000	0.44 ± 0.0201	0.59 ± 0.0270	1.22 ± 0.0559
1:2000	0.34 ± 0.0155	0.50 ± 0.0229	1.11 ± 0.0508
1:4000	0.25 ± 0.0114	0.42 ± 0.0192	1.31 ± 0.0600
1:8000	0.21 ± 0.0096	0.44 ± 0.0201	0.90 ± 0.0412
1:10000	0.18 ± 0.0082	0.37 ± 0.0169	0.85 ± 0.0389
1:20000	0.16 ± 0.0073	0.25 ± 0.0114	0.68± 0.0311
1:40000	0.12 ± 0.0057	0.22 ± 0.0101	0.30 ± 0.0137

Table S4. Enzyme-linked immuno sorbent assay showing the reaction of samples from different hosts using ToLCNDV-Rg-CP-Pab

Sl. No.	Hosts	OD value at 405 nm (Mean of 3 replications)		
		Buffer	Healthy	Infected
1.	Ridge gourd	0.25 ± 0.0115	0.30 ± 0.0137	1.41 ± 0.0646
2.	Watermelon	0.25 ± 0.0115	0.29 ± 0.0133	0.79 ± 0.0362
3.	Cucumber	0.25 ± 0.0115	0.29 ± 0.0133	0.97 ± 0.0445
4.	Pumpkin (Yellow mosaic)	0.25 ± 0.0115	0.28 ± 0.0128	0.84 ± 0.0385
5.	Bitter gourd	0.25 ± 0.0115	0.31 ± 0.0142	1.21 ± 0.0554
6.	Bottle gourd	0.25 ± 0.0115	0.40 ± 0.0183	1.05 ± 0.0481
7.	Snake gourd	0.25 ± 0.0115	0.30 ± 0.0137	1.02 ± 0.0467
8.	Pumpkin (Leaf curl)	0.25 ± 0.0115	0.26 ± 0.0119	0.94 ± 0.0312
9.	Chilli	0.25 ± 0.0115	0.23 ± 0.0105	0.27 ± 0.0124
10.	Capsicum	0.25 ± 0.0115	0.23 ± 0.0105	0.26 ± 0.0119
11.	Cow pea	0.25 ± 0.0115	0.37 ± 0.0170	0.38 ± 0.0174
12.	French bean	0.25 ± 0.0115	0.33 ± 0.0151	0.35 ± 0.0160

Table S5. Validation of DAC-ELISA by detection of ToLCNDV in plant samples collected from various fields in South India

Sl. No.	Location	Detection Techniques	
		PCR	DAC-ELISA OD_{405nm}
1	UHS Campus, Bagalkot, Karnataka	+	0.984 ± 0.0451
2	Badami, taluk, Bagalkot district, Karnataka	+	0.725 ± 0.0332
3	Jamakhandi, Jamakhandi taluk, Bagalkot district, Karnataka	+	0.813 ± 0.0373
4	Sanganakeri, Gokak taluk, Belagavi district	+	1.221 ± 0.0560
5	Athani, Athani taluk, Belagavi district	+	1.214 ± 0.0556
6	Ghataprabha, Gokak taluk, Belagavi district	+	1.011 ±0.0463
7	IIHR, Hesarghatta. Bengaluru, Karnataka	+	1.234 ±0.0565
8	Rajanakunte, Bengaluru north taluk,	+	1.089 ±0.0499
9	Doddaballapura, Bengaluru rural district, Karnataka	+	0.984 ±0.0451
10	GKVK campus, Bengaluru, Karnataka	+	0.725 ±0.0332
11	Basapur, Navalagund, taluk, Karnataka	+	0.913 ±0.0418
12	Rajoor, Yelburga taluk, Karnataka	+	0.721 ±0.0330
13	Bhatrahalli, Kolar taluk, Karnataka	+	1.412 ±0.0647
14	Hallikere, Maddur taluk, Mandya district	+	1.211 ±0.0555
15	Sindhuvalli, Nanjangudu taluk, Mysore district, Karnataka	+	1.134 ±0.0520
16	Koratakere, Sira taluk. Tumkur district, Karnataka	+	1.189 ±0.0545
17	Muddebihal, Muddebihal taluk, Vijapura district, Karnataka	+	1.211 ±0.0555
18	Vijayapur, Vijayapur taluk, Vijapura district, Karnataka	+	1.134 ± 0.0520
19	Poragi, Hindupur taluk. Anthapur district, Andhra Pradesh	+	1.089 ±0.0499
20	Bagalur, Hosur taluk. Salem district, Tamil Nadu	+	0.992 ±0.0455
21	Healthy Control	-	0.200 ±0.0153
22	Buffer control	-	0.380 ±0.0252

Table S6. Primers used in the present study for amplification of ToLCNDV coat protein gene

Name of the Primer	Sequence (5’-3’)	Restriction sites	Product (nt)	Reference
CCP-F	GCC(C/T)AT(G/A)TA(T/C)AG(A/G)AAGCC(A/C)AG	-	575	Wyatt and Brown, 1996
CCP-R	GG(A/G)TT(AGT)GA(G/A)GCATG(TAC)GTACATG	-		
CP-F	TAC<u>GGATCCATATGA</u>TGATGTCGAAGCGACCAGCA	*BamH*1 and *Nde*I	771	Present Study
CP-R	TAG<u>AAGCT</u>TTTAATTTGTGACCGAATC	*Hind*III		

*Nucleotides in underline represent the *BamH*I and *Nde*I restriction sites in the forward primer and *Hind*III restriction site in the reverse primer.

Table S7. GenBank accession numbers of selected begomovirus sequences from database used in this study for analysis of DNA-A component and coat protein

Sl. No.	Name of the Begomoviruses	Acronym	Accession No.
1	Tomato leaf curl New Delhi virus-[India:Karnataka:Belagavi:Ridge gourd:2014]	ToLCNDV-[IN:Kar:Bel:Rid:14]	-
2	Tomato leaf curl New Delhi virus-[India:Karnataka:Chettahalli:Ridge gourd:2015]	ToLCNDV-[IN:Kar:Chel:Rid:15]	KT426905
3	Tomato leaf curl New Delhi virus-[India:New Delhi:Ridge gourd:2010]	ToLCNDV-[IN:ND:Rid:10]	HM989845
4	Tomato leaf curl New Delhi virus-[Pakistan:Ridge gourd:2008]	ToLCNDV-[PK:Rid:08]	EF620534
5	Tomato leaf curl New Delhi virus-[Thailand:Cucumber:2007]	ToLCNDV-[TH:Cuc:07]	AB330079
6	Tomato leaf curl New Delhi virus-[Indonesia:Central Java:Cucumber:2008]	ToLCNDV-[ID:Cen:Cuc:08]	AB613825
7	Tomato leaf curl New Delhi virus - [Bangladesh:Cucumber:2006]	ToLCNDV-[BG:cuc:06]	EF450316
8	Tomato leaf curl New Delhi virus - [India: New Delhi:Cucucmber:2012]	ToLCNDV-[IN:ND:Cuc:12]	KC545812
9	Tomato leaf curl New Delhi virus-[India:New Delhi:Pumpkin:2006]	ToLCNDV-[IN:ND:Pum:06]	JN129254
10	Tomato leaf curl New Delhi virus-[India:New Delhi:Pumpkin:2006]	ToLCNDV-[IN:ND:Pum:06]	AM286433
11	Tomato leaf curl New Delhi virus - [India:New Delhi:Pumpkin 2:2005]	ToLCNDV-[IN:ND:Pum2:05]	AM286434
12	Tomato leaf curl New Delhi virus-[Pakistan:Multan:Bitter gourd:2005]	ToLCNDV-[PK:Mul:Bit:05]	AM747291
13	Tomato leaf curl New Delhi virus-[Thailand:Bottle gourd:2007]	ToLCNDV-[TH:Bot:07]	AB368447
14	Tomato leaf curl New Delhi virus-[India:TamilNadu:cucurbits:2014]	ToLCNDV-[IN:TN:cuc:14]	KM275598
15	Tomato leaf curl New Delhi virus-[Thailand:Musk melon:2007]	ToLCNDV-[TH:Mus:07]	AB368448
16	Tomato leaf curl New Delhi virus-[India:Sponge gourd:2005]	ToLCNDV-[IN:Spo:05]	AY939926
17	Tomato leaf curl New Delhi virus-[Taiwan:Oriental melon:2007]	ToLCNDV-[TW:Ori:07]	GU180095
18	Tomato leaf curl New Delhi virus-[India:New Delhi:Ash gourd:2011]	ToLCNDV-[IN:ND:Ash:11]	JN208136
19	Tomato leaf curl Palampur virus - [India:Uttarpradesh:Muskmelon:2015]	ToLCPalV-[IN:UP:MM:15]	KY564204
20	Tomato leaf curl Palampur virus - [India:Varanasi:Pumpkin:2008]	ToLCPalV-[IN:Var:Pum:08]	GQ225738
21	Tomato leaf curl Palampur virus - [Pakistan:Faiziabad:Bittergourd:2010]	ToLCPalV-[Pak:Fai:BG:10]	FN555137
22	Tomato leaf curl Palampur virus - [Iran:Jiroft:Melon:2007]	ToLCPalV-[IR:Jir:Mel:07]	EU547683
23	Tomato leaf curl Palampur virus - [Iran:Khash:W9P:Citrullus lanatus:2010]	ToLCPalV-[IR:Kha:W9P:Wat:10]	JF501728
24	Tomato leaf curl Palampur virus - [Iran:Jiroft:T69P:Cucumis sativus:2008]	ToLCPalV-[IR:Jir:T69P:Cuc:08]	JQ825226
25	Tomato leaf curl Palampur virus-[India:Allipur:Cucumber :2010]	ToLCPalV-[IN:All:Cuc:10]	HQ848383
26	Tomato leaf curl Palampur virus - [Iran:Jiroft 8:T58P:Cucumber:2007]	ToLCPalV-[IR:Jir8:T58P:Cuc:08]	FJ660431
27	Tomato leaf curl Palampur virus - [Iran:Jiroft 5:T51X:Cucumber:2007]	ToLCPalV-[IR:Jir5:T51X:Cuc:08]	FJ660432
28	Tomato leaf curl Palampur virus - [Iran:Kerman:T8X:Cucumber:2007]	ToLCPalV-[IR:Ker:Cuc:07]	FJ660433
29	Tomato leaf curl Palampur virus - [Iran:Kahnooj:T9X:Cucumber:2007]	ToLCPalV-[IR:Kah:T9X:Cuc:07]	FJ660434
30	Tomato leaf curl Palampur virus - [Iran:Jiroft 4:T6X:Cucumber:2007]	ToLCPalV-[IR:Jir5:T6X:Cuc:08]	FJ660436
31	Tomato leaf curl Palampur virus - [Iran:Jiroft 9:T7X:Cucumber:2007]	ToLCPalV-[IR:Jir9:T7X:Cuc:08]	FJ660437
32	Tomato leaf curl Palampur virus - [Iran:Jiroft 1:T55X:Cucumber:2008]	ToLCPalV-[IR:Jir1:T55X:Cuc:08]	FJ660444
33	Tomato leaf curl Palampur virus - [Iran:Jiroft:T13X:Cucumis melo:2006]	ToLCPalV-[IR:Jir:T13X:Me:06]	JF501719
34	Tomato leaf curl Palampur virus - [Iran:Jiroft:T61X:Cucumber:2008]	ToLCPalV-[IR:Jir:T61X:Cuc:08]	JF501723
35	Tomato leaf curl Palampur virus - [Iran:Jiroft:T5X: Cucumber:2007]	ToLCPalV-[IR:Jir:T5X:Cuc:07]	JF501724
36	Tomato leaf curl Palampur virus - [Iran:Iranshahr:M4P:Cucumis melo:2009]	ToLCPalV-[IR:Ira:M4P:Mel:09]	JF501725
37	Tomato leaf curl Palampur virus - [Iran:Jiroft:K1P:Cucurbita pepo:2009]	ToLCPalV-[IR:Jir:K1P:Squ:09]	JF501727
38	Tomato leaf curl Palampur virus - [India:Palampur:Pumpkin:2008]	ToLCPalV[IN:Var:Pum:08]	FJ931537
39	Squash leaf curl China virus-[China:Squash:2003]	SLCCNV-[CH:Squ:03]	AB027465
40	Squash leaf curl China virus-[Vietnam:Squash:2004]	SLCCNV-[VN:Squ:04]	AF509743
41	Squash leaf curl China virus-[Vietnam:Squash:2004]	SLCCNV-[VN:Squ:04]	AF509741
42	Squash leaf curl China virus-[India:Coimbatore:Pumpkin:2004]	SLCCNV-[IN:Coi:Pum:04]	AY184487
43	Squash leaf curl China virus-[India:Lucknow:Pumpkin:2004]	SLCCNV-[IN:Luc:Pum:04]	DQ026296
44	Squash leaf curl China virus-[India:Varanasi:Pumpkin:2004]	SLCCNV-[IN:Var:Pum:04]	EU573715
45	Squash leaf curl Philippines virus-[Philippines:Pumpkin:2008]	SLCPHV-[PH:Pum-08]	AB085793
46	Squash leaf curl Philippines virus-[Taiwan:Pumpkin:2005]	SLCPHV-[TW:Pum:05]	DQ866135
47	Squash leaf curl China virus-[Pakistan:Lahore:Zucchini:2004]	SLCCNV-[PK:Lah:Zuc:04]	AM286794
48	Chilli leaf curl-[Oman:Tomato:2016]	ChiLCV -[OM:Tom:16]	HE806437
49	Chilli leaf curl-[Oman:Tomato:2014]	ChiLCV -[OM:Tom:14]	KF229718
50	Chilli leaf curl-[India:Gujarat:Gandhinagar:Chilli:2014]	ChiLCV-[IN:GJ:Gan:Chi:14]	KM880103

51	Chilli leaf curl-[Oman:Watermelon:2017]	ChiLCV -[OM:WM:17]	KX787939
52	Chilli leaf curl-[Oman:Salalah:Petumia:2012]	ChiLCV -[OM:Sal:Pet:12]	HF968755
53	Chilli leaf curl-[Oman:Salalah:Petumia:2014]	ChiLCV -[OM:Sal:Pet:13]	HF968756
54	Chilli leaf curl-[Oman:Barka:Tomato:2014]	ChiLCV -[OM:Bar:Tom:14]	LN680633
55	Chilli leaf curl-[Oman:Barka:Chilli:2013]	ChiLCV -[OM:Bar:Chi:13]	HG969263
56	Chilli leaf curl-[Oman:Barka:Chilli:2016]	ChiLCV -[OM:Bar:Chi:16]	HG969264
57	Chilli leaf curl-[Oman:Barka:Chilli:2016]	ChiLCV -[OM:Bar:Chi:16]	HG969265
58	Chilli leaf curl-[India:Hariyana:Sonipat:Mint:2015]	ChiLCV-[IN:HR:Sont:Mint:15]	KM921669
59	Chilli leaf curl-[Oman:Barka:Tomato:2016]	ChiLCV -[OM: Bar:Tom:16]	HG969200
60	Mung bean yellow mosaic virus [India:New Delhi:Urdbean:2011]	MYMV-[IN:ND:Urd:11]	JQ398669
61	Mung bean yellow mosaic virus [India:TamilNadu:Madhurai:Beans:2015]	MYMV-[IN:TN:Mad:Bean:15]	AJ132575
62	Mung bean yellow mosaic virus [India:TamilNadu:Blackgram:2016]	MYMV-[IN:TN:Bg:16]	KP784665
63	Mung bean yellow mosaic virus [Pakistan:Faizalabad:Mungbean:2013]	MYMV-[PK:FZ:Mung:13]	FM208836
64	Mung bean yellow mosaic virus [India:Uttarpradesh:Varanasi:Frenchbean:2011]	MYMV-[IN:UP:Var:Fre:11]	KC019303
65	Mung bean yellow mosaic virus [India:Uttarpradesh:Varanasi:Frenchbean:2011]	MYMV-[IN:UP:Var:Fre:11]	KC019304
66	Mung bean yellow mosaic virus [India:New Delhi:Cow pea:2003]	MYMV-[IN:ND:Cow:03]	AF481865
67	Mung bean yellow mosaic virus [India:New Delhi:Frenchbean:2002]	MYMV-[IN:ND:Fre:02]	AY099066
68	Mung bean yellow mosaic virus [India: Uttarpradesh:Kanpur:Freanchbean:2010]	MYMV-[IN: UP:Kan:Fre:10]	GU591171
69	Mungbean yellow mosaic India virus - [India:Varanasi:Dolichos:2005]	MYMV-[IN:Var:Dlo:05]	AY547317
70	Mungbean yellow mosaic India virus - [India: New Delhi:Pigeonpea:2017]	MYMV-[IN:ND:PP:07]	KX363947
71	Mungbean yellow mosaic India virus - [India: Asssam:Guwahati:Cowpea:2017]	MYMV-[IN:AS:Guw:Cow:17]	KY556679

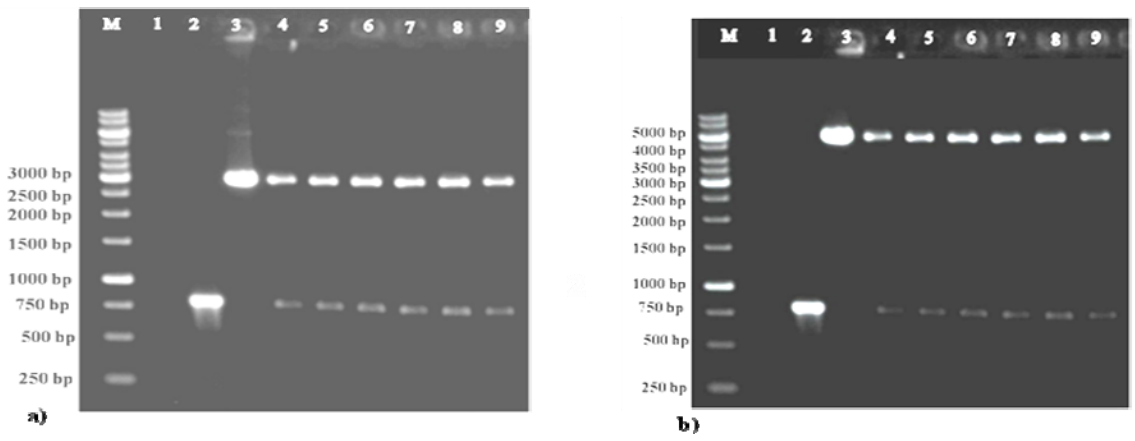

Figure S1. Agarose gel photograph showing restriction digestion of recombinant pBluescript IIKS(+)-ToLCNDV-Rg isolate-CP with *BamH*I and *Hind*III restriction endonucleases (a) and Restriction digestion of recombinant pET22-NH-ToLCNDV-Rg-CP with *Nde*I and *Hind*III restriction endonucleases (b). a) Lane M: 1kb DNA marker, Lane 1:Water control; Lane 2:Positive control; Lane 3:Undigested pBluescriptIIKS(+)-ToLCNDV-CP-Rg; Lanes 4-9:*BamH*I and *Hind*III restricted recombinant plasmid (pBluescriptIIKS(+)-ToLCNDV-CP-Rg); b) Lane M: 1 Kb DNA marker; Lane 1:Water control; Lane 2:Positive control; Lane 3:Undigested pET22-NH-ToLCNDV-CP-Rg; Lanes 4-9:*Nde*I and *Hind*III digested recombinant plasmid pET22-NH-ToLCNDV-CP-Rg.

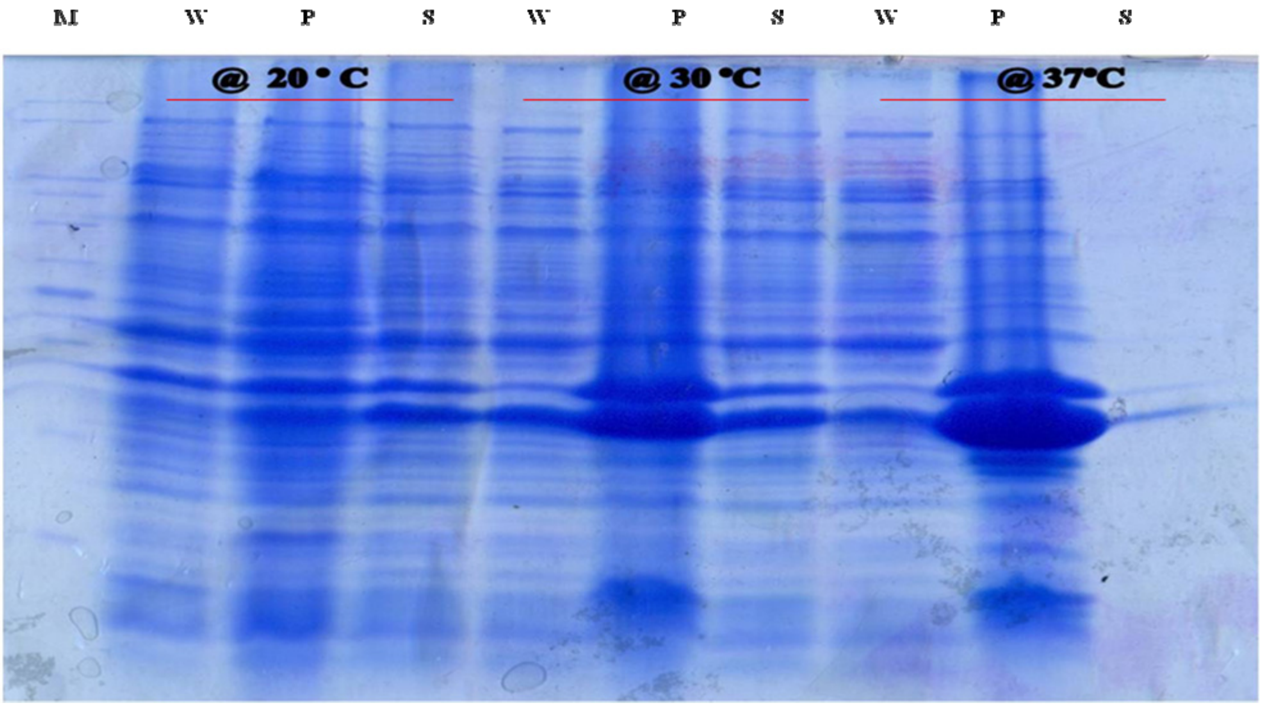

Figure S2. Expression of ToLCNDV-Rg-CP in *E. coli* at different concentrations of IPTG and incubation temperatures. Lane M: Protein marker; Lane 1: *E. coli* BL-21 cells;Lanes 2-7: Induction at 20 °C with IPTG at 0.0, 0.05, 0.1, 0.3, and 0.5 mM concentrations; Lanes 7-11:Induction at 30 °C with IPTG at 0.0, 0.05, 0.1, 0.3, and 0.5 mM concentrations;Lanes 12-16:Induction at 37 °C with IPTG at 0.0, 0.05, 0.1, 0.3, and 0.5 mM concentrations.

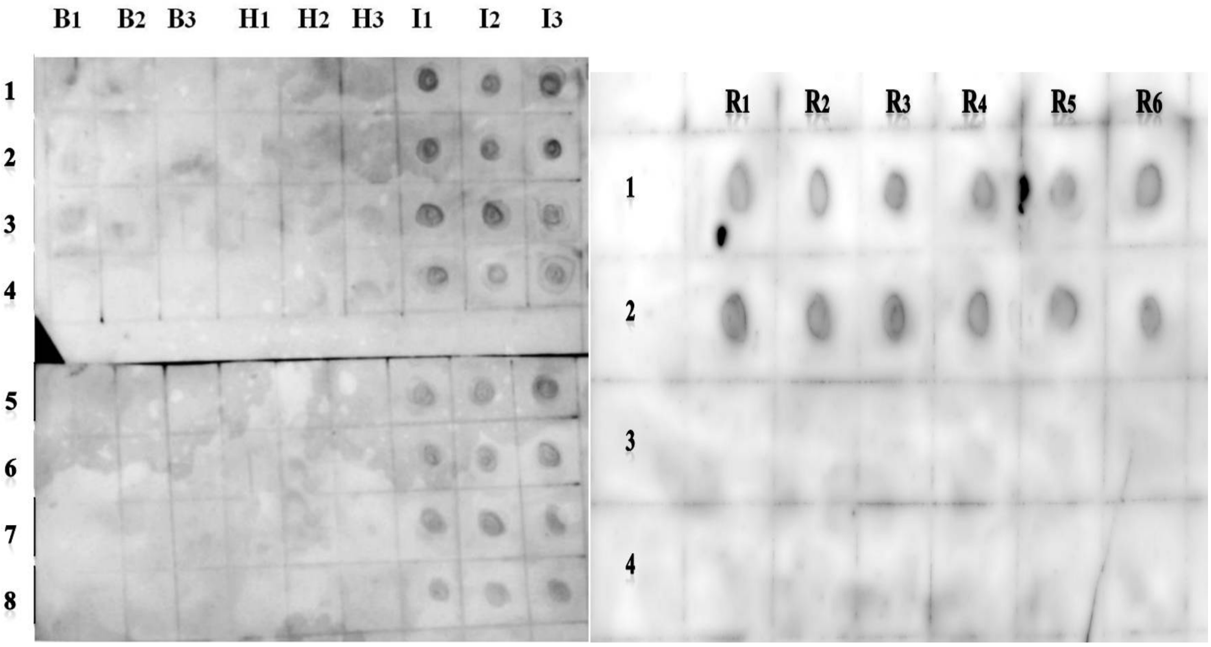

Figure S3. Dot immuno binding assay (DIBA) with different dilutions of ToLCNDV-Rg-CP-Pab tested with ToLCNDV-Rg-CP. a) B: Buffer control; H: Healthy control; I: Infected samples; Row 1:1:100; Row 2:1:500; Row 3: 1: 1000; Row 4: 1: 2000; Row 5:1:4000; Row 6:1:8000; Row 7:1:10000; Row 8: 1:20000; b) Row 1: Recombinant protein; Row 2: Ridge gourd infected sample; Row 3:Ridge gourd healthy sample; Row 4:Negative control (Buffer); R1; R_2; R_3; R_4; R_5 and R_6 are replications.

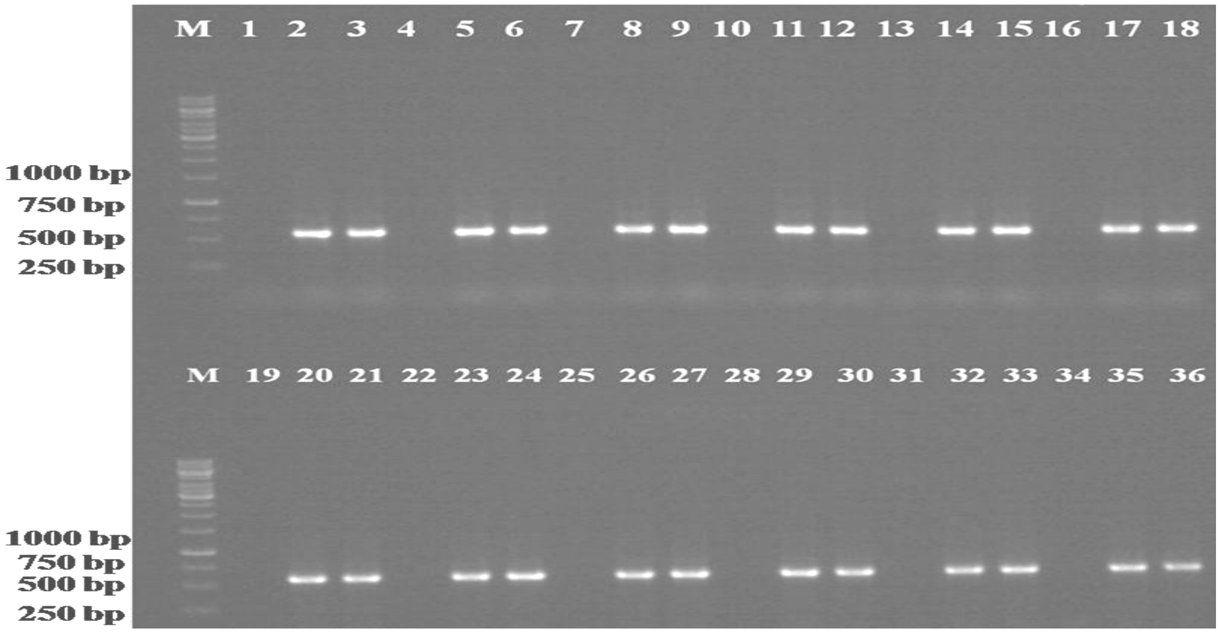

Figure S4. Agarose gel showing PCR amplification of core CP of begomoviruses from various infected host plants. M: 1 kb DNA marker; Lane 1:Healthy ridge gourd; Lane 2, 3:Infected ridge gourd; Lane 4:Healthy watermelon; Lane 5, 6 :Healthy watermelon; Lane 7: Healthy cucumber; Lane 8, 9:Infected cucumber; Lane 10 : Healthy pumpkin; Lane 11, 12 : Infected pumpkin; Lane 13:Healthy bitter gourd; Lane 14, 15 :Infected bitter gourd; Lane 16:Healthy bottle gourd; Lane 17, 18: Infected bottle gourd; Lane 19:Healthy sponge gourd; Lane 20, 21:Infected sponge gourd; Lane 22:Healthy tomato; Lane 23, 24:Infected tomato; Lane 25: Healthy chilli; Lane 26, 27: Infected chilli; Lane 28: Healthy capsicum; Lane 29, 30:Infected capsicum; Lane 31:Healthy cowpea; Lane 32, 33:Infected cowpea; Lane 34:Healthy beans; Lane 35, 36: Infected French bean.

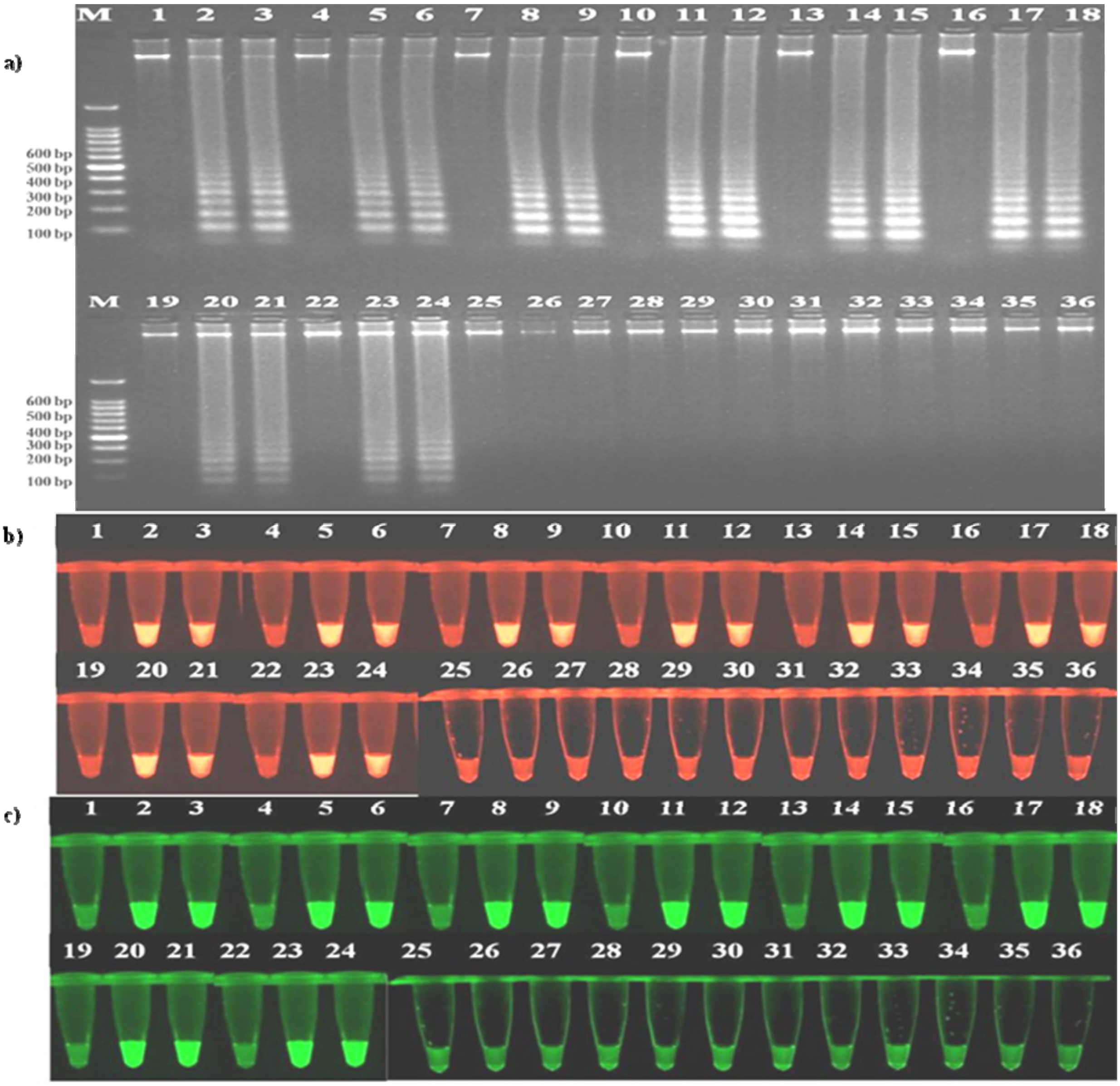

Figure S5. LAMP amplification of begomoviruses from various crops suspected to be infected with begomovirus and its visualization on a) 2 % agarose gel b), EtBr and c) VeriPCR dyes. M: 100bp DNA marker; Lane 1:Healthy ridge gourd; Lane 2, 3:Infected ridge gourd; Lane 4:Healthy watermelon; Lane 5, 6 :Infected watermelon; Lane 7:Healthy cucumber; Lane 8, 9 :Infected cucumber; Lane 10 :Healthy pumpkin; Lane 11, 12 :Infected pumpkin; Lane 13: Healthy bitter gourd; Lane 14, 15 :Infected bitter gourd; Lane 16: Healthy bottle gourd; Lane 17, 18: Infected bottle gourd; Lane 19:Healthy sponge gourd; Lane 20, 21:Infected sponge gourd; Lane 22:Healthy tomato; Lane 23, 24;Infected tomato; Lane 25:Healthy chilli; Lane 26, 27: Infected chilli; Lane 28:Healthy capsicum; Lane 29, 30:Infected capsicum; Lane 31:Healthy cowpea; Lane 32, 33:Infected cowpea; Lane 34:Healthy French bean; Lane 35, 36:Infected French bean.
